# Development of an intervention to increase HPV vaccine uptake in Colombia

**DOI:** 10.1186/s40814-025-01609-5

**Published:** 2025-05-31

**Authors:** Veronica Cordoba-Sanchez, Mariantonia Lemos, Sherri Sheinfeld Gorin

**Affiliations:** 1 Facultad de Psicología, Universidad de San Buenaventura, Carrera 56C N° 51-110, Medellín, Colombia; 2 Programa de Psicología, Escuela de Artes y Humanidades, Universidad EAFIT, Carrera 49 No. 7 Sur — 50, Medellín, Colombia; 3 University of Michigan School of Medicine, Joint Appointment, The School of Public Health, The University of Michigan, Ann Arbor, MI USA

**Keywords:** HPV vaccination, Cervical cancer, Human papillomavirus, Complex intervention, Behavior change

## Abstract

**Background:**

The Global Strategy for the Elimination of Cervical Cancer as a Public Health Problem proposed a goal of 90% coverage for HPV vaccination in girls between 9 and 14 years of age by 2030. However, despite the efficacy and safety of the vaccine, its coverage in Colombia remains low. The aim of this paper is to provide a detailed description of the creation of a protocol for an intervention to increase HPV vaccine uptake among vaccine-eligible children and adolescents in a school setting.

**Methods:**

This intervention development protocol was co-created with nine teachers, five nurses, nine parents, and seven girls in two workshops. Using structured worksheets, the transcripts of the workshops were integrated with the behavioral diagnosis resulting from the COM-B model and the intervention options from the Behavior Change Wheel.

**Results:**

The findings suggest that participants want to receive information through different means, according to their age, such as messages and videos from experts on viruses for parents, games and workshops for girls, and testimonials for all. The protocol “Superprotegidas” integrates such strategies.

**Discussion:**

The co-creation workshop identified the unique information sources and messages among stakeholders in this community. Basing the behavioral analysis on the COM-B model and the interventions on the Behavior Change Wheel undergirds the planned subsequent randomized clinical trial in a larger population.

**Supplementary Information:**

The online version contains supplementary material available at 10.1186/s40814-025-01609-5.

## Key messages regarding feasibility

What uncertainties existed regarding the feasibility?

- While the problem of vaccine hesitancy was conceptualized in a prior study, it was essential to determine the most effective content and delivery format for increasing HPV vaccine uptake among girls requiring parental consent, including the best approach to inform parents. Additionally, assessing the feasibility of implementing a behavioral intervention in a school setting remained a key consideration.

What are the key feasibility findings?

- In this intervention development study, we found that co-designing the intervention with parents, girls, teachers, and the municipal vaccination team was a promising approach to improving HPV vaccine uptake. Participants in the co-creation workshops expressed a preference for receiving information through diverse formats, including written and oral educational messages, testimonial videos, games, and workshops.

What are the implications of the feasibility findings for the design of the main study?

- The feasibility and acceptability of the intervention will now be tested in a feasibility study. Key considerations for the next study include collaborating with schools to recruit unvaccinated girls and their parents, using vaccine uptake as the primary outcome while identifying key secondary outcomes, and conducting qualitative interviews to assess acceptability.

## Background

Cervical cancer is the fourth most common cancer among women worldwide [1]. In 2020, per 100,000 women, Colombia had an age-standardized incidence rate of 14.9 and an age-standardized mortality rate of 7.43 [2]. Both rates are higher than those for cervical cancer in the rest of the world. In November 2020, the World Health Organization launched the Global Strategy for the Elimination of Cervical Cancer as a Public Health Problem, which proposes three goals to be achieved by 2030: 90% coverage for the complete HPV vaccination schedule in girls and adolescents, 70% of women receiving a highly accurate screening test by age 35 and again at age 45, and treatment for 90% of women with cancerous lesions [3].

Regarding the first goal, despite the efficacy and safety of the vaccine, its coverage remains low. By 2020, it was estimated that only 6% of all countries in the world had reached a coverage of more than 90% in the final dose, and globally, the coverage for the final dose reaches only 15% [4]. In Colombia, the estimated coverage for the first dose is 39% and 11% for the final dose [2].

Several explanations have been proposed to explain this low coverage (see summary [5]).

The HPV vaccine has unique characteristics that may generate potential communication problems at the time of its implementation, for example, it is relatively new, has been aimed at girls and preadolescents, it is injectable, it may have a high cost, and it originally required two or three doses, with a long interval between them. It protects against a little known sexually transmitted infection and against a cancer that can manifest itself many years after infection. Additionally, even if the vaccination schedule has been completed, this does not exempt the need for cervical cancer screening measures [6].

Preventive medical visits — including routine vaccines — decrease substantially as children become adolescents, which reduces the opportunity to vaccinate in health centers. Further, many primary care clinics do not include the HPV vaccine in their vaccination workflows, so the HPV vaccine is not administered with other childhood and adolescent vaccines (such as meningitis). When eligible children and adolescents are seen by the provider but do not receive a recommendation, this creates a “missed opportunity” for HPV vaccine administration [7–9]. Lack of healthcare provider recommendation is a widely recognized barrier to HPV vaccine uptake.

HPV vaccine delivery generally requires parental consent until the age of majority. It is easier to obtain this consent in health centers where parents and daughters are present simultaneously than in schools [10]. Parents are another central influence on the minor’s HPV vaccination decision. Parental lack of knowledge about the vaccination [11], low-perceived susceptibility to cervical cancer, and the sexualization of the HPV vaccine are key barriers to the uptake decision [12, 13].

The vaccination crises that have occurred in countries such as Denmark [14, 15], Japan [16], Austria [17], and Colombia [18–20] have encouraged myths, misinformation, and concerns about safety and possible adverse effects of the HPV vaccine. Similar to the rapid spread of misinformation during the height of the covid-19 pandemic, media coverage and spread of these large-scale psychogenic responses to the HPV vaccine could be termed an “infodemic” [21].

While studies of predictors to HPV uptake have burgeoned over time, the quality of the evidence has ranged from moderate to low, according to a recently published comprehensive review [22]. Importantly, high-quality randomized clinical trials of behavioral interventions to increase HPV vaccine uptake are needed in middle- and low-income countries in which the burden of HPV is high and vaccination coverage is lower than in high-income countries. Since vaccination is a behavior, in the absence of effective behavioral interventions for change, patients are vulnerable to misinformation, with a potential erosion of trust in healthcare for cancer prevention [23].

The development of evidence for behavior change rests on the ORBIT model that describes a sequence of structured phases similar to those for the development of pharmacological treatments: Phase 1: Define-refine, Phase 2: Preliminary evaluations, Phase 3: Efficacy study, and Phase 4: Effectiveness study [24]. We recently conducted the Phase I trial among girls aged 9–15 who were students in Medellin schools and their parents; using a mixed-methods approach, we identified the reasons for the low vaccination uptake [19].

In Colombia, HPV vaccine has been school based since its introduction in 2012, and coverage was adequate until the crisis of 2014 [20]. Schools have been considered an attractive setting for the delivery of new immunizations because of their ability to reach large numbers of children in a short period of time [25]. As vaccination requires signed parental consent, educational interventions are necessary for students and parents to understand the importance of HPV vaccination and to promote the return of consent forms [26, 27].

The aim of this paper is to provide a detailed description of the development of a behavioral intervention protocol for future testing in a feasibility study and a randomized controlled trial to increase HPV vaccine uptake among vaccine-eligible children and adolescents. We describe the process of developing an intervention for girls, through co-creation workshops, among adolescents aged 9–14 years and their parents and guardians in a Colombian municipality.

## Methods

### Setting

The intervention will be conducted in Envigado (Colombia), a town that is part of Metropolitan Area of the Aburra Valley. This is the second largest urban area in Colombia, with a population of over four million. Envigado currently has 14 public schools that serve students from first through 11th grade. The HPV vaccination program is administered by school-based nurses from the Municipal Health Secretariat who visit the schools twice a year and provide one dose of the HPV vaccine to girls ages 9–17 with parental written consent. Students can also get the vaccine from the health provider for free.

### Protocol development

Two co-creation workshops were conducted between June and October 2022, according to the guidelines proposed by the *Co-Creation Handbook* [28]. The workshops were conducted in two schools in the town of Envigado. One of the schools was public and the other private (to examine different contextual factors), and they were selected because both had been contacted through a previous study about HPV vaccine hesitancy [19].

Participants were nine teachers (five women and four men), five nurses from the Municipal Health Secretariat (three women and two men), nine parents (all women), and seven unvaccinated girls between 9 and 14 years of age (daughters of the participants’ parents). The participants were convened by the academic coordinators of the schools (teachers and unvaccinated girls and one of their parents), and the nurses were directly invited by the researchers. The sample size was determined based on principles of qualitative research, specifically purposive sampling, to ensure representation of key stakeholder groups relevant to the intervention, including educators, healthcare professionals, parents, and adolescents. This approach was chosen to capture a diverse range of perspectives and insights critical for the co-creation process.

Each of the co-creation workshops was conducted by one of the researchers (V. C. S.) with the support of research assistants (psychology students) in a classroom of the participating schools; each workshop lasted approximately 1 h and a half, and each workshop followed a standardized protocol that included the following: (1) introduction (15 min), (2) construction (30 min), (3) plenary (30 min), and (4) closing (15 min), as described below:

1. Introduction

We began with a brief motivational introduction; we shared information about the problem of low HPV vaccination uptake and the importance of the participation of each of the actors affected by the problem. We used an ice breaker to begin the discussion, in which each participant took one end of a skein of wool, introduced him/herself (name and type of participant), and threw the skein to another participant until a spider web was formed, representing the importance of each participant in weaving a solution to the problem presented.

2) Construction

Participants were divided into teams, and each team was made up of different types of participants (a girl and her respective parent, a teacher, and a nurse). Each team was assigned two findings from the previous study [19] in simple language, with a question (e.g., parents believe that vaccinating their daughters is an action that demonstrates their intention to care for and protect them. How can this data help us get more parents to vaccinate their daughters? Parents associate HPV with early sexual debut and are therefore hesitant to have their daughters vaccinated. How can we ensure that this concern does not prevent parents from having their daughters vaccinated?); each team was given a sheet of paper, markers, pens, and post-its to write their group’s responses to the posed questions. We allotted 30 min for this activity, and refreshments were provided during this phase of the workshop.

3) Plenary

The chairs in the room were arranged around a round table; each team came to the front with their sheets of paper to present the solutions they had found to the assigned topic. The other participants were encouraged to make suggestions and to nurture the solutions presented. This phase was recorded, and photographs were taken of the material written by the participants with their permission and then collected for further analysis.

4) Closure

During this phase, participants’ additional questions were answered, and everyone was thanked for their contributions to the collective construction of the solution to the problem presented.

### Theoretical model

The COM-B model, including the Behavioral Change Wheel (BCW), was used to undergird the workshop; this model has been widely used for behavioral trials, including for understanding vaccine uptake [29]. The model highlights three important factors that can influence behavior: capacity, opportunity, and motivation. Capacity refers to an individual’s mental and physical ability to engage in a particular activity, opportunity relates to external physical and social factors that allow a behavior to occur, and motivation encompasses the conscious and unconscious cognitive processes that influence and drive the behavior [30].

After defining the sources of behavior, following the COM-B model, according to the BCW, you can define the intervention functions, which can be delivered through behavior change techniques (BCTs), an active component of the intervention to change behavior designed to alter or redirect causal processes that regulate behavior [31]. Every step of the process followed the structured worksheets from the BCW [32].

### Analysis plan

The groups’ example responses from the co-creation workshops, as well as the transcribed audio recordings, were de-identified and checked for accuracy. We conducted a thematic analysis as part of a descriptive, qualitative approach [32]. Themes were discussed, described in summary memos, and linked to illustrative quotes. Some illustrative quotes were lightly edited for readability.

The behavioral diagnosis, that is, the assessment of influences on the desired target [18], HPV vaccination, was assessed using the COM-B model. We linked the diagnosis to the intervention options from the BCW using a thematic analysis of the structured worksheets [32], the group’s example responses, and the transcribed audio recordings. The BCTs included the following: information on health consequences, material incentive, credible source, instruction on how to perform a behavior, adding objects to the environment, demonstration of the behavior, social support, information on emotional consequences, and self-reward.

Two of the authors (V. C. S., M. L.) discussed the intervention options together to evaluate their connection to the behavioral diagnosis and their appropriateness. Intervention appropriateness was determined using the APEASE criteria (Affordability, Practicability, Effectiveness and cost-effectiveness, Acceptability, Side effects/safety, and Equity) [31].

### Ethical considerations

Before starting the co-creation workshops, all adult participants gave their written consent, the parents gave their authorization to the girls, and the girls gave their informed assent. All participants were provided with materials to answer the written questions which were photographed and stored, and the interactions in the workshop were recorded and later transcribed for later analysis. This project was approved by the Institutional Review Board of Universidad EAFIT.

## Results

We analyzed the transcripts from the workshops to design the subsequent intervention protocol.

Participants expressed a lack of information; both adolescents and parents in both workshops expressed a need for accurate information about cervical cancer (location of the affected organ, severity of the disease, prevalence, and need for prevention) as well as about HPV (mode of transmission, prevalence) and the HPV vaccine.

We came to the conclusion that it is through education in the vaccination system that parents can access information and know what the benefits and consequences are. It is the only way they will be able to make the decision to vaccinate their daughters (Mother, private school).

They also mentioned that training (both face to face and remote) and other communication technologies popular with adolescents (such as games and chats) are most appropriate to transmit this information:

The best way to get the information to the girls would be with talks with games. Something like a game that helps us to know more about human papilloma and a teaching kit that suddenly explains how to avoid it, how we can be more protected from human papilloma (Girl, public school).

We should have a chat, for example, for parents, to inform parents what they have to do (Girl, public school).

Workshops that involve parents, girls and boys, and pedagogical material on the topic. I came up with the idea of doing some training sessions that I saw online, in which we click on next, next or next and you get animated information, so to speak, about the human papilloma (Mother, private school).

Regarding the vaccine, they stated that they were interested in the types of viruses against which it protects safety and efficacy. They preferred that this specific medical information be shared by experts (gynecologists, immunologists).

As well as gender equity, the Health Secretariat can also come from the fourth grade to talk to them about papilloma, because it is different for a psychologist to come than for a gynecologist to come and explain to parents and children and explain the benefits of this (mother, public school).

An immunologist would be excellent and would counteract the fears of the parents and they would be aware with this and scientific studies (mother, public school).

Similarly, they suggested that desexualizing the vaccine is important, that is, emphasizing that the vaccine does not incite the initiation of sexual activities but is a preventive measure taken beforehand.

It is very common for parents to associate the fact of vaccinating with opening the door to sexuality, but it is important to clarify that these are very different things and that in the end this is also part of the information that the girl can receive not only at school but also at home, so it is important to clarify and talk about the greater good at stake, which is to avoid infection and in the distant or not distant future to avoid contracting a disease that could lead to death, so we need to aware parents and clarify this type of information (Teacher, public school).

I think it is important for parents to keep in mind that this is not a contraceptive but that we have to aware parents that we must educate ourselves so that they remove the taboo that if I vaccinate my daughter I am encouraging her to sexuality, we have to know very systematically how to transmit this information (Mother, public school).

They stated that the emotional benefits of vaccination (the peace of mind of protecting their daughter) should be highlighted.

The fact of feeling secure, of feeling calm gives an internal sensation of being able to develop well and when a person, I do not know if it has happened to you, that when one has a flu or an illness one does not approach others, one speaks to them as if from a distance, one is internally coerced instead to take care of oneself and I believe, I also believe that psychologically and emotionally it gives a stability and a balance. Obviously nobody is exempt from an illness, we can all get sick, but I think that this vaccination and knowledge also gives one a certainty and security in the social environment. (Teacher, private school)

Several participants mentioned that a very appropriate way to transmit the information would be through various testimonies, from people who have been vaccinated and from people who have had (HPV-related) cancer.

Testimonies of women who have perhaps gone through the disease and have come out ahead or people, women or girls, who have been vaccinated and have gone through it and say: look, I got vaccinated, I went through this, I did this, and it was not what we thought, it went well, I did well. I think that these testimonies are very useful for raising awareness. (Teacher, private school)

The testimony is given from the girl who applied it and will say I felt bad I got dizzy I got this, but someone told me that all vaccines have their reactions there have always been reactions but that any reaction that gives one is important for protection. Even an adult person who did not get the vaccine because they did not explain it to her at the time, nor did she see the need or think that she could get the virus and she did not get the injection and she could have contracted the virus and explain that they should not do what she did, and another person who did get the vaccine and all her life has had her periodic exams and nothing has happened to her (Mother, public school).

Make a parallel of testimonies: for example, if someone has not been vaccinated against papillomavirus and listens to someone else who has, how she felt, if it went well or badly or if nothing happened, the testimony motivates her to get vaccinated (Girl, public school).

Participants suggested that the heightened awareness of COVID-19 could present an opportunity for HPV vaccination:

We can take advantage of all this COVID-19 to raise awareness of the fact that there is a scientifically proven drug that can prevent a serious disease and that can give us, in one way or another, the chance to preserve life, and we talk about what is also important at a statistical level, it is not the same to talk about COVID-19, which many already consider as just another flu, as it was considered exactly 2 years ago when they told us that vaccination would take 12 or 13 months and that this vaccination will be gradual, it will be limited and that today we have a vaccine that can obviously avoid the possibility of contracting cancer in our girls and women (Teacher, public school).

It is also a health and self-care issue and I believe that COVID taught us that self-care immediately replicates in co-responsibility and that taking care of me is good to take care of others (Teacher, private school).

Additionally, a nurse from the municipal vaccination team pointed out the importance of involving all stakeholders in an intervention to increase HPV vaccinations:

I have been in the extramural vaccination program of the municipality for 5 years, one of the work fronts of this program is the application of this HPV vaccine. This vaccine is full of prejudices, but prejudices from us as parents and it is important to know that we all have to get involved to be able to make these coverages go up. By everyone, I mean parents and institutions (Nurse).

Finally, a nurse from the vaccination team emphasized that schools are the best setting to reach age-eligible girls for the HPV vaccine:

When we started the campaign five years ago, it was directed at the parents, and we summoned them to the schools, but they did not attend. Then, the schools told us that parents did not come to the parent-teacher meetings, so they were not going to come to an awareness meeting about a vaccine that is surrounded by prejudices because people believe in them. So, we began to raise awareness among girls, and vaccination coverage was triggered at that time in the schools.

Our analyses led to a set of intervention strategies (see Table 1).

**Table 1:** Strategies for girls and adolescents and their parents based on the COM-B model

Source of behavior	Factors to address in the intervention	Intervention function	BCTs	Strategy
Psychological capability	Lack of information Relationship between HPV and cervical cancer	Education	Information on health consequences Material incentive Credible source	For girls and adolescents: A pedagogical and playful workshop session with a 3D model of the female reproductive system, gamification through a knowledge contest, and an infographic with summary information about cancer, HPV, and vaccination For parents: A letter and three text messages with information on HPV, cervical cancer, efficacy, and safety of the vaccine and one text message with the video of an expert opinion
Physical capability	How to fill a consent form	Training	Instruction on how to perform a behavior	For girls and adolescents: During the pedagogical and playful workshop session, participants will receive information about how to get the vaccine at the school, they should take an informed consent form home, give it to the parents to sign it, and return it to the school. The leader will demonstrate how to complete the informed consent form; some data must be filled out on the first page and signed on the back. Strategies will be generated to facilitate timely return of the signed form For parents: On the back page of the letter, there will be instructions about how to fill and sign the informed consent
Physical opportunity	Vaccination on the schools	Enablement	Adding objects to the environment^a^	For girls and adolescents: The vaccination day will be held at the school For parents: A reminder text message will be sent the day before vaccination for them to remember to sign the informed consent
Social opportunity	Looking other people get vaccinated Trust in traditional institutions	Modelling Persuasion	Demonstration of the behavior Credible source Social support	For girls and adolescents: During the workshop session, there will be a short story with the testimony of a girl and her father who were hesitant about the vaccine but after being informed decided to uptake the vaccine For parents: A text message will be sent with a video from *Colombian League Against Cancer*, with an expert talking about the efficacy and safety of the vaccine Starting from the first text message, the participants will have the support of the interventionist to solve doubts about the HPV vaccine through chat
Reflective motivation	Association of HPV with sexuality	Persuasion Modelling	Information on emotional consequences Information on health consequences Social support (unspecified)	For girls and adolescents: During the workshop session, there will be a short story about a cervical cancer survivor who desires to protect her daughters from that disease with the HPV vaccine For parents: A letter where it is stated that HPV vaccination should be initiated well before the onset of sexual activity for the body to have time to develop defenses against the virus One text message with one story about a father and his daughter who were hesitant about the HPV vaccine and decided to uptake it after receiving information and one text message with a video about a cervical cancer survivor who desires to protect her daughters from that disease with the HPV vaccine
Automatic motivation	Respect for the personal decision to be vaccinated (girls and adolescents) The vaccine as an act of care (parents)	Persuasion	Self-reward	For girls and adolescents: During the workshop session, it will be stated that HPV vaccination is the best way to be super protected against cervical cancer For parents: A letter where it is stated that HPV vaccination will protect the daughter against cervical cancer

^a^This will be done by the nurses of the vaccination team of the Municipal Health Secretariat. The resultant intervention (see Appendix) was reviewed by four health professionals based on the TIDier checklist in Spanish [33]

## Discussion

The objective of the present study was to develop an intervention protocol with the collaboration of stakeholders in a co-creation workshop, in preparation for a feasibility study and a randomized controlled trial. The findings suggest that participants want to receive information through different means, according to their age, such as messages and videos from experts in viruses for parents, games and workshops for girls and adolescents, and testimonies for all of them. They also considered the importance of desexualizing the vaccine and the emotional (e.g., peace of mind) benefits of vaccination.

The analysis of the co-creation workshops with stakeholders to increase HPV vaccination uptake in girls and adolescents yielded both traditional strategies such as the delivery of written information and the implementation of educational activities. The findings also revealed more novel communication approaches such as multimedia messages through chat services. Further, the study’s findings have identified which BCT can be implemented to increase vaccination.

The strategies described in this paper coincide with those raised in other studies reviewed [22, 34], regarding which strategies were more effective to increase vaccination. These include the following: the use of a 3D clinical model of the female reproductive tract to train girls and adolescents [35], interactive education sessions [36, 37], the use of videos [38], the use of written educational materials [27, 39], text messages [40, 41], reminder letters to parents [42, 43], and training about the informed consent that parents sign to authorize vaccination in minors [26]. While the informed consent document was not described in detail by participants in the co-creation workshop, both this recent paper [25] and a recent government policy document [44] emphasized the importance of education about the HPV vaccine to increase parental informed consent.

Additionally, the Cochrane review [45] examined interventions to increase vaccination rates in adolescents. The review found that multicomponent interventions, which include at least one educational session, repeated contacts, incentives, and other strategies, have moderate evidence to improve vaccine delivery compared to traditional practices. Adolescents educated about vaccination are better able to communicate with and influence their parents regarding the specific benefits of the HPV vaccine, including its safety and efficacy. Increasing both parental and adolescents’ knowledge about the risk of viral infection, the prevention of cervical cancer and genital warts, sexually transmitted infections, and their prevention, as well as dosages and age of vaccination, are key. Since the intention to vaccinate among parents is a strong predictor of vaccination behavior, changing the attitudes and beliefs of both parents and adolescents toward vaccination can generate synergies to improve uptake [12, 27, 34].

Following the recommendations proposed for the development of behavioral interventions in the ORBIT model [23], the novelty of this protocol lies in the behavioral analysis through both the COM-B model [19] and the BCW framework [32] to identify what changes are required to increase HPV vaccination uptake in a specific community. Further, the perspectives of stakeholders obtained through co-creation [28] designed concrete strategies that show promise for increasing HPV vaccination uptake in a LMIC context.

### Strengths and limitations of the study

The co-creation workshops only included health professionals from the extramural vaccination team; HMO medical service personnel who could also offer vaccinations are not included in this study. As a qualitative study to develop an intervention protocol, it is exploratory and hypothesis-generating, so *per force*, small, with few self-selected participants.

This intervention is designed for extramural vaccination teams who are taking the vaccine to school settings, so generalizability to other settings requires further adaptation. However, schools have been found effective in reaching large numbers of age-eligible children and adolescents. The more effective approaches have included in-school vaccinations, particularly in settings with regular schooling through adolescence [27, 46]. Further, in schools, nurses, teachers, and other professionals (even other parents and adolescents) can encourage HPV vaccine uptake.

It is important to note that the Colombian Ministry of Health recently updated the HPV vaccine dosage guidelines, now including 9-year-old boys and administering only one dose to girls aged 9 to 17 [47]. However, this study was conducted prior to the dosage change. Future studies should include boys in the analysis to fully capture the impact of the new guidelines.

## Conclusion

By involving stakeholders, it is possible to generate interventions that are feasible and understandable to the final recipients [5]. The process of co-creation [28] allowed us to anticipate the demands of future users. We found that the perspectives of students and teachers are fundamental to this process. These co-creation findings have demonstrated the optimal intervention strategies for vaccination behavior change.

An intervention protocol to increase the HPV vaccine, focusing on reminders and information delivered though several strategies, is particularly relevant to the recent IARC announcement about the efficacy of a single dose of a quadrivalent vaccine [48]. A single dose can facilitate vaccination behavior by reducing the resources required for return visits.

In 2023, the Colombian government decided that this is the opportunity to immunize males aged 9 years [47], as has been done in other countries [49]. While this policy change came after this study was complete, new strategies, focused on communicating to young males and their parents, will likely be necessary. As found in other low- and middle-income countries that newly instituted vaccines for boys after those for girls, the policy could lead to new waves of reluctance to vaccinate [45].

The next step was to test, through a feasibility study [50], whether the intervention developed in the present study could help Colombia to reach the WHO policy target HPV vaccinations to children and adolescents.

## Supplementary Information

Supplementary Material 1: Appendix: Protocol.

## Data Availability

The anonymized data and materials are available from the first author (V. C. S.).
