# Supplementary material for: Development of an intervention to increase HPV vaccine uptake in Colombia
Source: Pilot Feasibility Stud. 2025 May 31;11:74. doi: 10.1186/s40814-025-01609-5 (PMC12125825; doi:10.1186/s40814-025-01609-5)
Supplement: Supplementary file 1 — Supplementary Material 1: Appendix: Protocol. [file 40814_2025_1609_MOESM1_ESM.docx]

**Appendix:** Protocol

# Brief name: Provide the name or a phrase that describes the intervention:

Superprotegidas: a strategy to increase HPV vaccination in girls and adolescents aged 9 to 15 years.

# Why: Describe any rationale, theory or objective of the essential elements for the intervention.

HPV vaccination is a complex behavior, WHO (2013) considers that the HPV vaccine has some characteristics that make it different from other vaccines aimed at children, among them are that it is a vaccine aimed at girls and preadolescents, it is injectable, of recent invention, requires two doses and a space of several months between doses, protects against a little known sexually transmitted infection, prevents against a cancer that manifests itself many years after infection and its application does not eliminate the need for screening.

Based on the behavioral conceptualization of the problem published in Cordoba-Sanchez et al. (2022) based on the COM-B model [30], it is necessary to address the different sources of behavior:

***Capacity****:* participants perceive susceptibility to cervical cancer and HPV, are aware of the need for vaccination; however, most lack information on the subject.

The intervention will provide written information to the caregiver and the recipient (girl/adolescent), will provide testimonials through a written story and several videos of vaccinated girls, of women who had cervical cancer.

***Opportunity:*** There is a relationship between likelihood of being vaccinated, having a medical recommendation, seeing others vaccinated and environmental opportunity.

The intervention will have videos of doctors and the messages delivered will use the social norm by saying that all parents will authorize the vaccination of their daughter and a hashtag will be promoted for girls and adolescents to post on their social networks when they are vaccinated.

Likewise, the intervention will be made before the municipal vaccination is going to be carried out at the educational institution.

***Motivation:*** There is concern about the safety and efficacy of vaccination, the relationship between HPV and the onset of sexual activity could be related to the reluctance to be vaccinated.

The intervention will have videos of experts and testimonies in which the safety and efficacy of vaccination will be addressed and the messages delivered will emphasize that vaccination should be done before the onset of sexual life because this will give the body time to develop defenses against the virus.

# What (materials): Describe any physical or informational materials used in the intervention, including those provided to participants or used in the delivery of the intervention or in the training of intervention providers. Provide information on where the materials can be accessed (e.g., online appendix, URL).

Room with chairs, computer graphics, chart, pens and notebooks for girls and adolescents. Cell phone with Internet access to send messages via Whatsapp.

# What (procedures): Describe each of the procedures, activities and/or processes used in the intervention, including enabling or supportive activities.

The intervention group will be the dyad of caregivers with their respective daughters.

**1. Intervention to girls and adolescents**

The daughters will receive the following workshop conducted by a psychologist in which a story will be told about a girl and her father and how they go from the girl not being vaccinated to being vaccinated.

***Workshop***

***Moment 1: Icebreaker activity***

*All participants should introduce themselves and tell how they are called, how old they are and what they like most about their body (eyes, hair) or what my body can do (dancing, running, playing).*

*Facilitator gives back: we all like our bodies to be well and to function well.*

***Moment 2: History***

*Ana is a 13-year-old teenager and her father's name is Jorge. Ana likes to play sports, play piano, study and math. Her father says that she is the queen of the house, she is everything for her family and they are very excited about her.*

*Ana's school went to vaccinate her against the human papillomavirus (HPV) and she did not get vaccinated, but she saw that her classmates did. She and her dad have some questions about how she would benefit from getting the vaccine.*

*The team from the Colombian League Against Cancer came to her house and told them that getting the HPV vaccine was the best way to prevent cervical cancer. Cancer is a disease in which cells multiply out of control, can spread to other parts of the body, block the normal functioning of organs and cause death.*

*The HPV vaccine generates defenses in the body and works as a shield that protects the body so that the virus does not turn into cancer.*

*After hearing this information Ana and her dad decided that getting vaccinated was the best decision.*

***Moment 3: Gamification***

*The facilitator mentions that he is going to conduct a knowledge contest about a part of the body that only girls, adolescents and women have.*

*We are going to do a random game (a dice or tingo tango) to assign the turn, whoever gets a question and is given a small incentive. If it comes back and falls on the same person we continue with the person on the right.*

*The questions are:*

- 1. *What is that part of the body that only women, girls and adolescents have and that men do not have?*
  2. *Where is this organ (the uterus)?*
  3. *What is this organ (the uterus) used for?*
  4. *What diseases can you get?*
  5. *What is cervical cancer?*
  6. *What causes cervical cancer?*
  7. *How is the Human Papilloma Virus transmitted?*
  8. *What other diseases can Human Papillomavirus cause?*
  9. *How is it prevented (Vaccine)?*
  10. *How many doses of the vaccine should I get?*
  11. *Where should I go or what should I do to get the vaccine?*

*In each question the facilitator provides the information, expanding or clarifying it:*

*Location of the uterus: The pelvis and* *uses a 3D-Model of the female reproductive system to show the parts ( the cervix and the main body).*

*Function: gestation*

*Emphasis on the cervix and its function: It is a channel that communicates the vagina and the uterus, through which both menstruation and babies come out at the time of delivery.*

*Cervical cancer is a disease that can cause tumors that spread to other parts of the reproductive system, which may require surgical treatment and even cause death.*

*HPV: This cancer is specifically caused by a virus called Human Papilloma Virus, which is sexually transmitted. It is very frequent and once it enters the body it can stay there for a long time, damaging the cells little by little until it generates lesions.*

*Other diseases: It can also generate genital warts and other types of cancer such as genital anal and throat cancers.*

*Prevention: There is good news: there is a vaccine to avoid catching the virus. Two doses, 6 months apart*

*It is placed in the school or in the HMO*

*To get the vaccine at the school you must take the informed consent form home, give it to your parents and return it to the school. The informed consent form will be shown, and it will be taught that some data must be filled out on the first page and signed on the back and strategies will be generated to remember to give it to the parents and take it to school on the corresponding day.*

***Moment 4: Closure***

*Delivery of infographics in the form of a shield containing key information:*

*
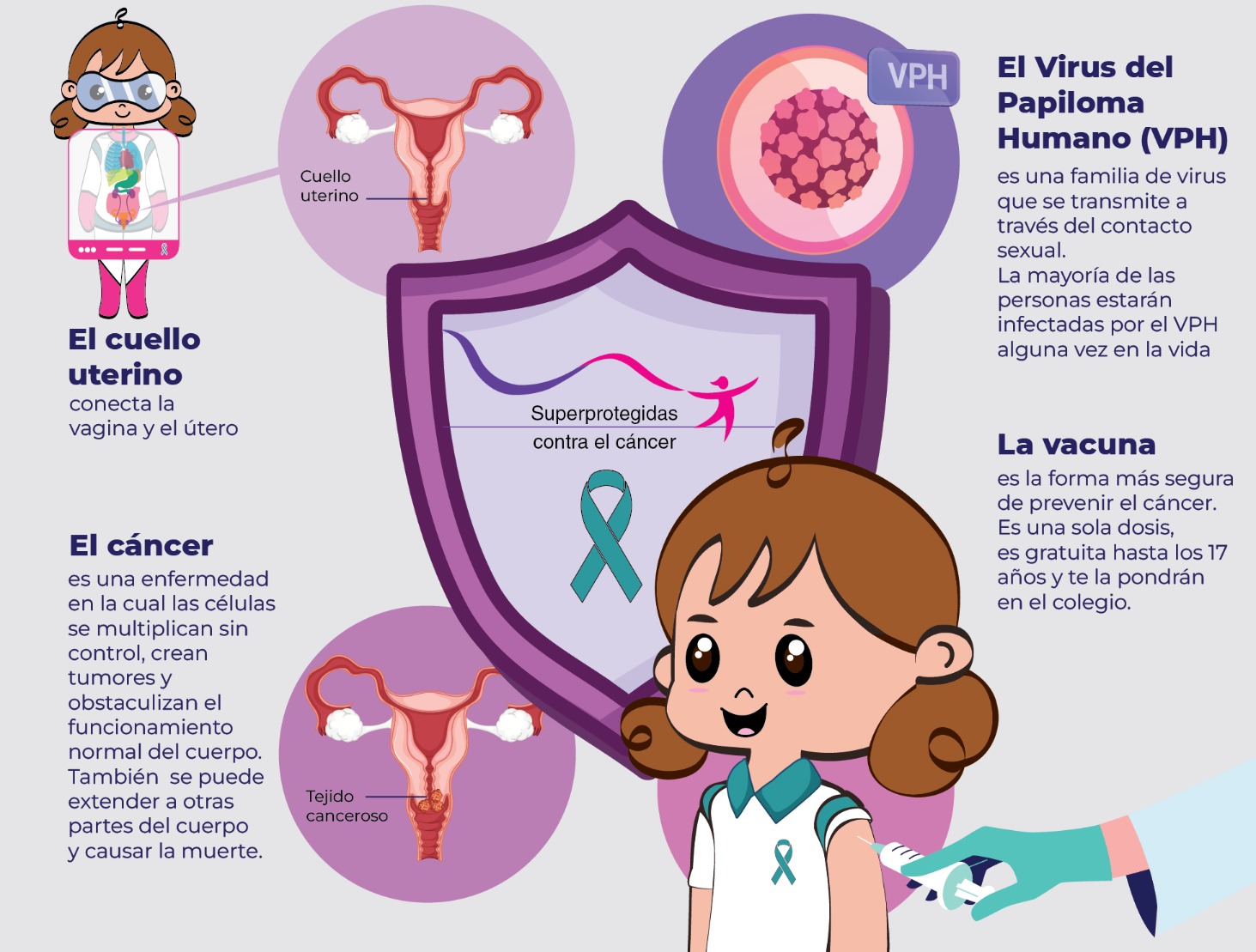
*

*The facilitator hands out the infographic and a letter for the parents, which has at the beginning a line for the name of the parent and at the end a line for the name of the child/adolescent and asks them to fill it out and give it to the parents.*

*Finally, the facilitator thanks the participants and ask them to post on social networks with the hashtag #SuperProtegida when they get vaccinated.*

**2. Intervention to parents**

Parents will in turn receive the following letter, the same the daughter was instructed to give them in the workshop:

*Dear Mom/Dad (parent’s name)__*

*We have great news for your family: Did you know that you can now protect your daughter against cervical cancer? The Human Papilloma Virus (HPV) is the main cause of cervical cancer and at the same time the most frequent sexually transmitted infection: 4 out of 5 people have been infected with HPV at some point in their lives and in Colombia there are about 4,700 new cases of cervical cancer each year.*

*The good news is that with a vaccine you can prevent your daughter from suffering from this disease as an adult. It is better to vaccinate girls long before they start their sexual life so that their organism develops defenses that protect them so that when they become women they do not develop cancer, that is why the vaccines are for girls from 9 years old and require two doses to work.*

*The vaccine is safe and effective and has been given to millions of children, adolescents and adults around the world. The Colombian League Against Cancer and the World Health Organization consider that vaccination is the best way to protect women against this cancer.*

*As a parent, you have a very important role in helping the health system protect your daughter. How can you do this? The municipal vaccination team will come to vaccinate your daughter and her classmates at school and will require your written authorization, so all parents will sign the Informed Consent that the girls will take home.*

*This way your daughter (daughters name)__ will be Super Protected against cervical cancer.*

In the back of the letter there will be instructions to fill the Informed Consent correctly:


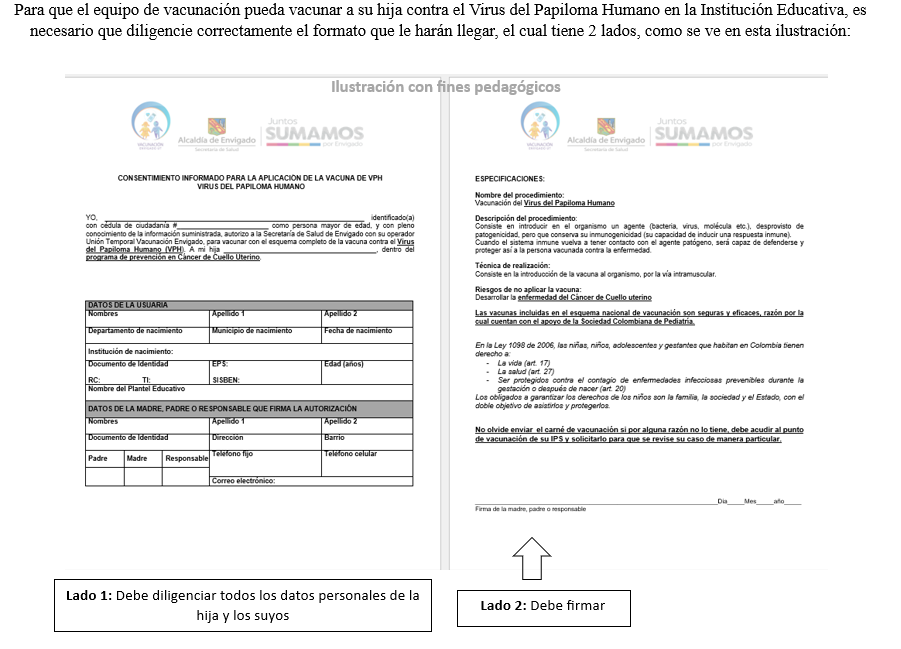


The week following the letter the following messages will be sent via whatsapp:

Dr. Nubia Muñoz, a physician from Cali nominated for the Nobel Prize in Medicine, highly recommends vaccination against HPV: https://www.youtube.com/watch?v=i9ZTivOgbKo

We can help you with any questions you may have about the vaccine, write us a message to: 3054208548

-Week 3, the following message will be sent via whatsapp

Ana is a 13 year old teenager and her dad's name is Jorge. Ana likes to play sports, play piano, study and math. Her dad says that she is the queen of the house, she is everything for her family and they are very excited about her.

Ana's school went to vaccinate her against the human papillomavirus (HPV) and she did not get vaccinated, but she saw that her classmates did. She and her dad have some questions about how she would benefit from getting the vaccine.

The team from the Colombian League Against Cancer came to her house and told them that getting the HPV vaccine was the best way to prevent cervical cancer. Cancer is a disease in which cells multiply out of control, can spread to other parts of the body, block the normal functioning of organs and cause death. In 2020, 4,742 new cases of cervical cancer were reported in Colombia.

The HPV vaccine generates defenses in the body and works as a shield that protects the body so that the virus does not turn into cancer.

After hearing this information Ana and her dad decided that getting vaccinated was the best decision.

- On week 4, the following message will be sent via whatsapp

Yineth is a cervical cancer survivor, check out her story and her daughters' stories: https://www.youtube.com/watch?v=dVe_4P1RZzo&t=1s

We can help you with any questions you may have about the vaccine, write us a message at: 3054208548

- On week 5, the following message will be sent via whatsapp

Here you can find all the information you need about cervical cancer prevention for your daughter: [*http://regalodevida.co/pageflip/padres.php*](http://regalodevida.co/pageflip/padres.php)

We can help you to solve any questions you may have, write us a message to: 3054208548

-On the day that the vaccination team raises awareness in schools, the following message will be sent:

Dear mom/dad

Today the vaccination team of the Health Secretariat visited your daughter's school and gave her an Informed Consent form. You must fill in the information on the front, sign it on the back and remind your daughter to take it to school tomorrow. Don't miss this great opportunity to give your daughter a future free of cervical cancer.

We can help you to solve any questions you may have, write us a message to: 3054208548

# Who provided: For each category of intervention provider (e.g., psychologist, nursing assistant), describe your experience, background, and any specific training provided.

The intervention will be carried out by a psychologist with experience in the subject, with a master's degree in Health Psychology Research, certified in the course *The Safety of the HPV Vaccine - Colombia Edition.*

# How (mode of delivery; individual or group): Describe the modes of delivery (such as face-to-face or by some other mechanism, such as internet or telephone) of the intervention and whether it was delivered individually or in a group.

A one-hour face-to-face group workshop for girls conducted at the educational institution .A letter sent to parents.

5 text messages sent to parents.

Opportunity to write to resolve questions via chat.

.

# Where: Describe the type(s) of location(s) where the intervention occurred, including any necessary infrastructure or relevant features.

Educational institution where children and adolescents study.

# When and how much: Describe the number of times the intervention was administered and over what period of time, including the number of sessions, their timing, and their duration, intensity or dosage.

A workshop session for girls.

Six parent communications: initial letter and reminder messages.

# Tailoring: In tailored interventions, not all participants receive an identical intervention. Was this intervention planned to be personalized, titrated, or tailored? [Yes/No]

There will be no adaptations of the intervention.

# 11 How well (planned): Fidelity refers to the degree to which an intervention occurred in the way the researchers intended. This item, and item 12, go beyond simple receipt of the intervention (such as how many participants received the intervention drug or exercises) and refer to "how well" the intervention was received or administered (such as how many participants took the drug/did the exercises, how much they took/did, and for how long).

The intervention will be carried out by the principal investigator of the project, during its development it is possible to think about the instruments that would be necessary to evaluate the fidelity in subsequent adaptations.
